# Biomimetic approach to strengthen the incisal fracture composite build-up: an in vitro study

**DOI:** 10.1186/s12903-023-03679-4

**Published:** 2024-01-08

**Authors:** Ganesh R. Jadhav, Priya Mittal, Siddharth Shinde, Mohammed A. Al-Qarni, Mohammed Al-Obaid, Shahabe Saquib Abullais, Marco Cicciù, Giuseppe Minervini

**Affiliations:** 1 Dept of Dentistry, AIIMS Nagpur, Nagpur, India; 2 Department of Conservative Dentistry and Endodontics, Swargiya Dadasaheb Kalmegh Smruti Dental College & Hospital, Nagpur, India; 3 Department of Orthodontics and Dentofacial Orthopedics, Bharati Vidyapeeth (Deemed to be University) Dental College and Hospital, Pune, Maharashtra India; 4 Department of Restorative Dental Sciences, College of Dentistry, King Khalid University, Abha, 61471 Saudi Arabia; 5 Department of Periodontics and Community Dental Sciences, King Khalid University, 61421 Abha, KSA Saudi Arabia; 6 Department of Biomedical and Surgical and Biomedical Sciences, Catania University, Catania, 95123 Italy; 7 Saveetha Dental College and Hospitals, Saveetha Institute of Medical and Technical Sciences (SIMATS), Saveetha University, Chennai, Tamil Nadu, India; 8 Multidisciplinary Department of Medical-Surgical and Dental Specialties, University of Campania “Luigi Vanvitelli”, Caserta, 81100 Italy

**Keywords:** Biomimetic, Composite resin, Debonding, Fiber-reinforced composite

## Abstract

**Objective:**

Incisal composite build-up shows a high failure susceptibility. The incorporation of fiber-reinforced composite (FRC) during composite restoration could improve its strength. Hence the study was planned to compare the effect of various positions of FRC on the strength of composite resin incisal build-ups.

**Methods:**

In maxillary incisors (n = 90), 3 mm of the incisal edge was cut and teeth were categorized into three groups based on the location and number of fibers used during incisal composite build-up - Group I: composite resin; Group II: composite resin and a single fiber palatally and Group III: composite resin along with two fibers palatally.

**Results:**

The data showed that group II had the maximum load-bearing values followed by group I and group III.

**Conclusion:**

Within the confines of our study, it can be concluded that the addition of FRC to the conventional incisal composite build-up increased the overall strength restoration. Such composite restoration reinforced with a single fiber on the palatal side showed the highest load-bearing capacity compared to two fibers reinforced and unreinforced composites. The common mode of failure in group I was in composite resin, in two fibers reinforced at fibers-composite junction, and in one fiber reinforced composite was in the remaining part of the tooth.

## Introduction

Traumatic dental injuries, common in children and adolescent populations, are caused by various factors like falls, road traffic accidents, sports injuries, assaults etc [1]. In permanent dentition, maxillary central incisors show higher predilection (around 65.65%) for such injuries by virtue of their position, projection and inappropriate lip enveloping. The primary goals in the management of such coronal tooth fractures are aesthetic and functional rehabilitation. Several factors influence the management of coronal tooth fractures such as the extent and pattern of fracture, restorability, concomitant soft tissue injuries, occlusion, aesthetics, finances etc. [2–9].

In such fractures, composite resin restoration is a treatment of choice due to its superior aesthetic property [10]. However composite restorations show a high failure rate (around 5%) due to their susceptibility to fracture [11, 12]. Moreover, only 50–60% of fracture resistance was obtained using composite build-up compared to intact incisors. Hence numerous attempts were made to improve the properties of composite resins [13]. The word ‘biomimetic’ indicates the biologically fashioned approach/material designed to imitate the nature [14, 15]. The biomimetic approach integrates the ideas from various disciplines such as materials science, biology, and bioengineering. The contemporary approach in conservative dentistry involves the use of minimally invasive treatment approach with bioinspired materials to achieve the best results.

An array of treatment approaches was attempted for the successful management of an uncomplicated crown fracture. The treatment strategies endeavored include the use of orthodontic bands, stainless steel crowns, pin retained composite restoration, and porcelain crowns [16]. However, these methods compromised the esthetics as well as tooth material. Hence the application of these techniques was not popular. The fractured fragment reattachment was proposed to be a biomimetic substitute for anterior teeth rehabilitation. However this technique pose a high risk of debonding in case of minor injuries [17]. hence the anterior restorative material should not only be esthetic and but also able to endure the impact forces during re-trauma [18]. In the past, attempts have been made to improve the load-bearing capacity of restoration by using different bonding systems and adhesive resins. One of such approaches involve the integration of nano-fillers or polyethene fibers in the composite to augment its mechanical properties. The development of fiber-reinforced composite (FRC) technology has led to substantial improvement in the physical properties of dental composite resin. FRC is composed of a thermoplastic polyethylene polymer that is widely applicable in many other fields [19]. In dentistry, FRC can be used in post-cores, space maintainers, splints, and removable prosthodontics [20]. Polymer chains in FRC offer superior impact resistance due to even force distribution [21, 22]. Previous studies found that composite resin reinforcement with fibers improved its fracture resistance [23–27]. It is non-invasive, cost-effective, time saving technology that protects restoration against fractures. Literature lacks studies on the fracture strength of FRC-reinforced incisal composite resin build-up.

Hence, it was hypothesized that fiber reinforced composite could improve the strength of composite restoration in incisal build-ups. Therefore, the study was planned to compare the effect of various locations fiber reinforcement on the strength of composite resin incisal build-ups.

## Materials and methods

The research protocol was sanctioned by the Institutional Ethical Committee (IEC) (IEC/10/2022/7). The samples used were extracted incisors and no humans were involved. Informed consent was obtained from the participants for the use of their teeth for research purposes.

### Sample size calculation

G Power (Version 3.1) software was used for sample size calculation. The sample size was found to be a minimum of 27 per group determined to achieve a 5% of Type I error, 80% of a power for an effective size of 0.5.

### Study design and specimens preparation

In this in-vitro experimental study, one hundred twenty freshly extracted human maxillary incisors were collected. Taking all aseptic precautions, calculus, bony debris, and soft tissue remnants were removed from the root surfaces using ultrasonic scalers 0.5% sodium hypochlorite was used for 15 min to disinfect teeth and stored in distilled water until further use. Teeth with wasting defects, cracks, caries, and previous treatments were excluded from the study (n = 9). Periodontal probe was used to select the teeth with similar dimensions (n = 94). The silicon index of each tooth’s crown was prepared so that it acts as a guiding template for the composite build-up. With the help of a surveyor, teeth were mounted at 45˚inclination into an acrylic block till the cemento-enamel junction. In all teeth, 3 mm of the incisal edge of was cut using a thin diamond cutting disc under water-air coolant. Periodontal probe was used to check the dimensions of the cut incisal surface for uniformity. Teeth that showed any visible pulp exposures or cracks were excluded from the study (n = 4).

### The prepared teeth (N = 90) were categorized randomly in three groups

Group I (control) (n = 30): Incisal edges were restored with Nanohybrid composite resin (Filtek™ Z250 Universal Restorative, 3 M, ESPE, USA).

Group II (n = 30): Incisal edges were restored with Nanohybrid composite resin (Filtek™ Z250 Universal Restorative, 3 M, ESPE, USA) and a single fiber (Ribbond, Seattle, WA, USA) on the palatal surface in the center.

Group III (n = 30): Incisal edges were restored with Nanohybrid composite resin (Filtek™ Z250 Universal Restorative, 3 M, ESPE, USA) and two fibers (Ribbond, Seattle, WA, USA) at different positions.

### Incisal edge build-up

For group I, the incisal margin of all teeth was beveled (about 0.5 mm) using a coarse flame diamond bur. The bevel region was selectively etched (30 s for the enamel and 15 s for the dentin) using a 32% phosphoric acid gel (Scotchbond™ Universal Etchant, 3 M, ESPE, USA) 1 mm beyond the bevel region. Subsequently, the gel was rinsed thoroughly with water and gently air-dried. A dentin bonding agent (Adper™ Single Bond 2 Adhesive, 3 M, ESPE, USA) was applied in 2–3 layers according to the manufacturer’s instructions and was polymerization using a LED light-curing unit for 20 s. The incisal part was restored with Nanohybrid composite resin (Filtek™ Z250 Universal Restorative, 3 M, ESPE, USA) and polymerized incrementally in two layers using a LED light curing unit for the 40s. With the help of previous dimensions of all teeth measured by using William’s graduated probe and the silicon index of an individual tooth, the crown lengths were adjusted to be the same length as that of the original length and the restorations were completed. Polishing and finishing of the composite restoration were done.

For group II, a small box-shaped cavity (1 × 2 × 1mm3) was prepared adjacent to the incisal edge on the mid-palatal surface of each tooth using a straight fissured. Beveling of preparation and etching were done in the same way as done in group I. 2 mm of the FRC (Ribbond, Seattle, WA, USA) was cut and stored in a bonding agent for 5 min in a dark place. The FRC was then gently inserted in the palatal box and extended 1 mm coronal to the fractured edge. Polymerization was carried out using a LED light-curing unit for 20 s. The composite build-up was completed in the same manner as it was done in group (I) For group III, two small boxed-shaped cavities (of similar dimensions as in Group II) were prepared 1 mm apart from each other on the palatal surface to incorporate two fibres. All other steps were followed as in group (II) All restored teeth were stored at 37 degrees centigrade in distilled water for 7 days before testing.

### Preparation of teeth for application of load under universal testing machine

Static load was applied to the restored teeth with a Universal testing machine at a speed of 1 mm/min. The acrylic block containing the restored tooth was tightly fixed upright to the base to provide 45 degrees angle between the palatal surface of the tooth and the loading tip (spherical − 2mm2). The load was applied at the mid-palatal surface adjacent to the incisal edge. The load event was registered until fracture for each tooth and the failure mode of each specimen was visually analyzed.

### Statistical analysis

The data were entered in a Microsoft Excel sheet, and SPSS software (version 19.0, Statistical Package for the Social Sciences Inc, Chicago, IL, USA) was used for statistical analysis. The mean load-bearing capacity of different test groups was represented as mean ± standard error of means (Table 1). The data were analyzed using the Kruskal-Wallis test of one-way ANOVA (for non-parametric data) followed by Dunn’s multiple comparisons tests. The p-value of ≤ 0.05 was considered statistically significant.

**Table 1:** Mean ± SE load bearing (N) capacity of three different groups confidence interval limits of three different groups

Groups	Mean ± SE	95% CI
**I**	**501.8 ± 21.45**	457.9-545.6
**II**	702.5 ± 20.66	660.2-744.7
**III**	520.6 ± 23.74	472.0-569.1

## Results

**Fig. 1:**
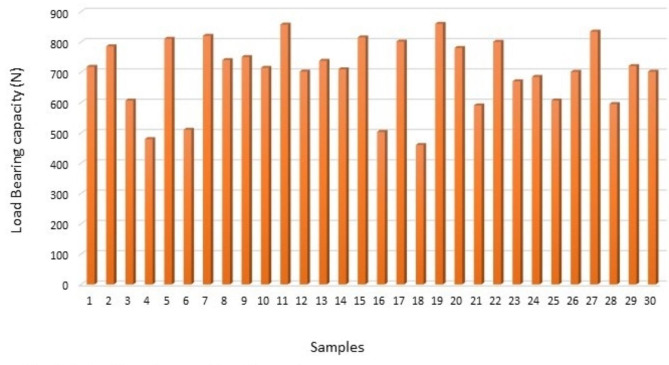
Bar graph representing load bearing capacity of group I

**Fig. 2:**
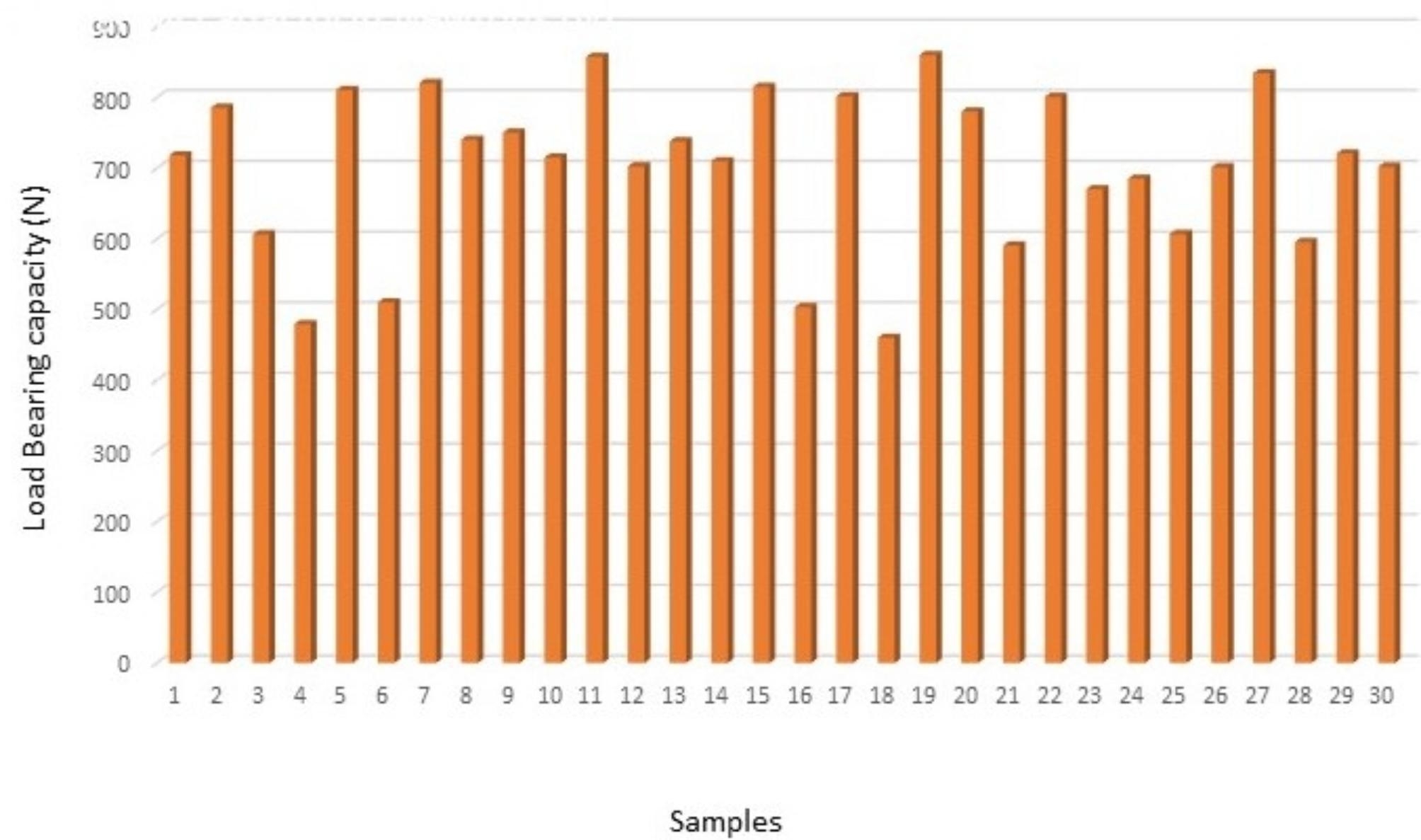
Bar graph representing load bearing capacity of group II

**Fig. 3:**
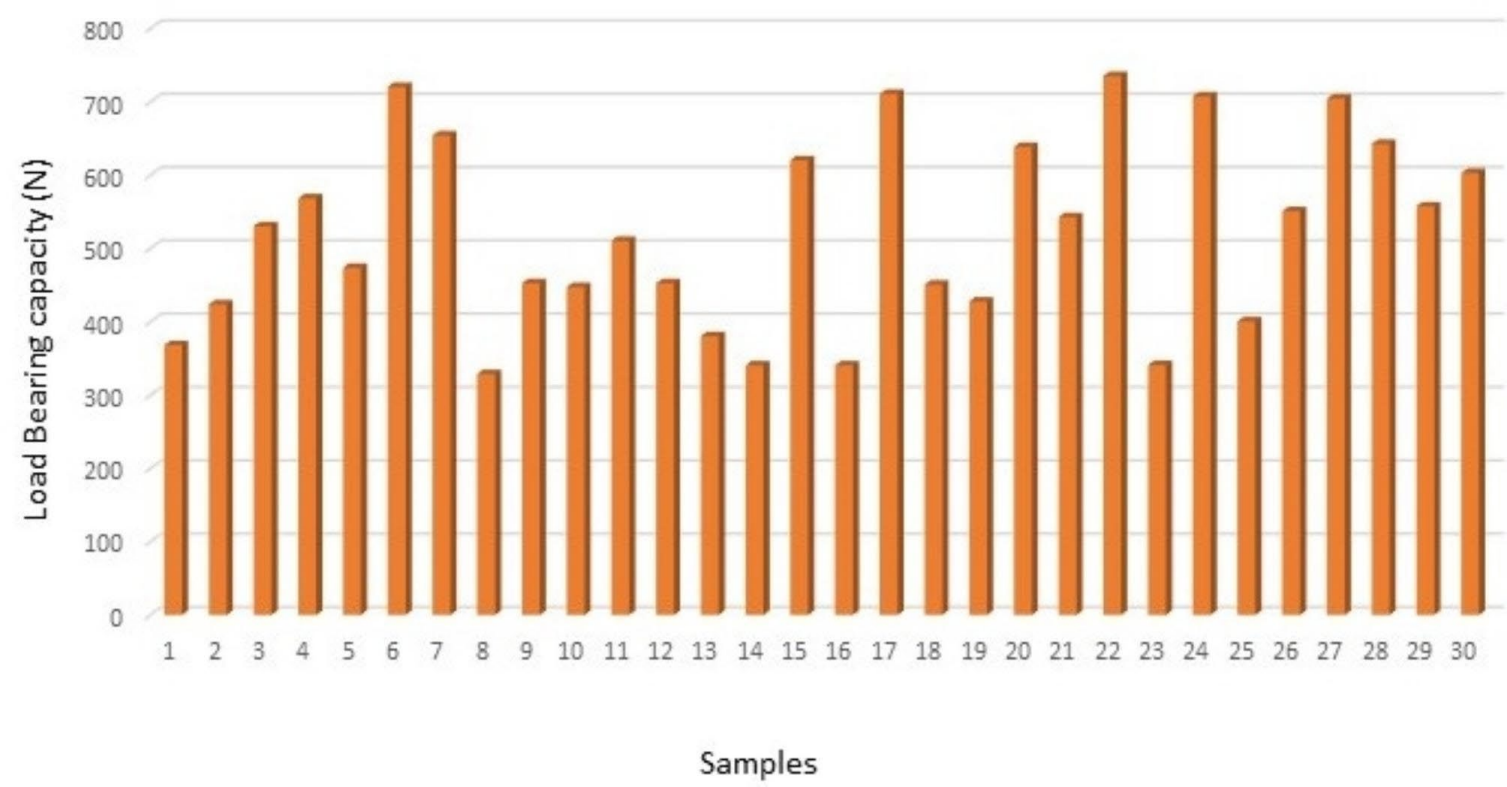
Bar graph representing load bearing capacity of group III

The load bearing capacity of all the samples from groups I, II and III are depicted in (Figs. 1, 2 and 3) respectively. The data showed that group II had the maximum load-bearing values followed by group I and group III (Table 1).

**Fig. 4:**
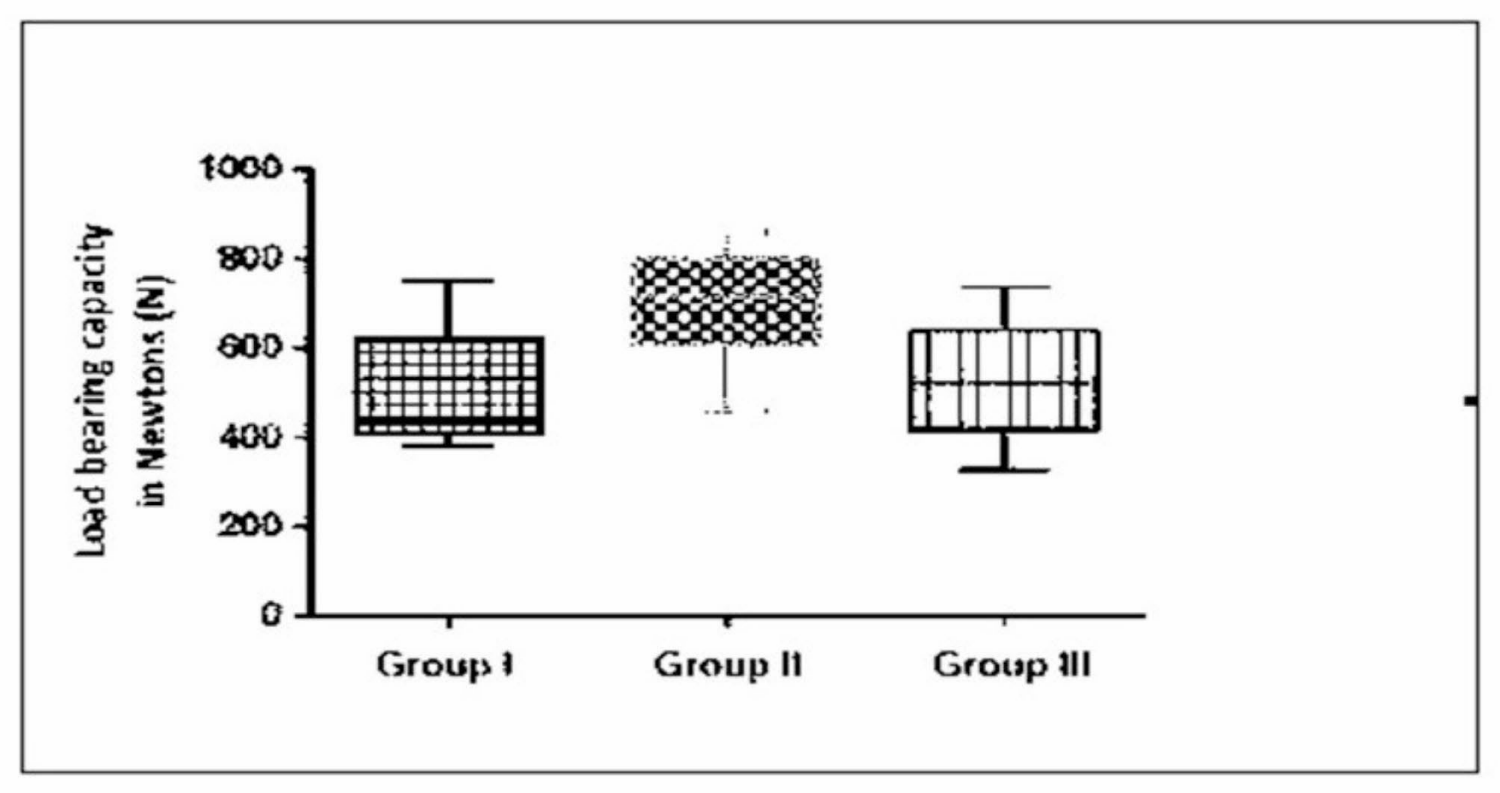
Box-and-whisker type of plot showing load bearing capacity of all the groups

In a box-and-whisker type of plot, the upper and lower lines of the box denote the upper and lower reading values respectively and the middle line in the box denotes the median of the sample (Fig. 4).

No statistical difference was found between the load-bearing values of groups I and III, while there was a statistically significant difference between the load-bearing values of groups II and III and groups I and II (Table 2). Minimum, Median and Maximum value for all groups is shown in Table 3. Most of the failures that occurred in group I was between the remaining part of the tooth and the restored edge. In group II, most of the failures were in the remaining part of the tooth. In group III, the most of failures were seen in the restored edges.

**Table 2:** Comparison of mean rank difference and statistical significance between all groups

Groups	Mean rank difference	p value
**I and II**	-34.17	0.0001*
**I and III**	-3.533	0.99
**II and III**	30.63	0.0001*

**Table 3:** Minimum, Median and Maximum value for all groups

Groups	No of samples	Minimum value (N)	Median value (N)	Maximum value (N)
**I**	30	380.4	435.9	754.2
**II**	30	460.2	716.6	860.0
**II**	30	328.5	520.1	735.0

## Discussion

The primary objective evaluated in this study was a comparative evaluation of the fracture resistance of the nano-hybrid composite resin restorations with and without FRC for incisal build-up. Uncomplicated anterior crown fractures are conservatively restored using the composite build-up. The main requirements of composite restorative material for incisal edge build-up are aesthetics and high fracture resistance. Hence constant advances were made in the field of adhesive dentistry to magnify its mechanical properties. Despite such advances, the composite resins may not fully restore the fracture resistance of the intact tooth and limit their use for large anterior long-term restoration. To overcome these shortcomings, FRC can be incorporated during the composite restoration. It is a silanized, bondable, pre-impregnated, plasma-treated, leno-woven, ultra-high molecular weight polyethylene fibers [28, 29]. Leno-weave is a special pattern of cross-linked threads that increases the durability, stability and shear strength of the FRC due to its high coefficient of elasticity [30]. The typical architecture permits its closed adaption to the outlines of a tooth [31]. The dense nodal junctures of the material decrease the likelihood of damage by evading the fiber displacement during handling. “Gas-plasma” treatment is done in FRC allowing a good chemical bond to composites along with reducing the microcracking [32]. It is a biocompatible, esthetic, translucent material that disappears within the composite, thus improving the esthetic outcome. For these reasons, FRC appears to be the ideal strengthening choice and hence FRC was chosen here to strengthen the composite.

In this study, the tooth was loaded at 45 degrees which created a relative angle of 135 degrees to the palatal surface that closely simulated the clinical scenario of the protrusive function. Group II showed a statistically significant higher load-bearing capacity compared to group I. These findings were similar to the findings of the previous study [33]. FRC-reinforced composite provides improved mechanical properties desirable for the incisal build-up by distributing the forces. This diminished the stress at the interface and created a larger bonding area that is helpful under repeated loading. Moreover, these fibers act as crack stoppers to improve the strength of a composite [34]. Restoration’s success depends on the cohesiveness between the fibers and the surrounding resin matrix, which should ensure uniformity of stress transfer from the matrix to the fibers. The quantity of composite resin amid the fibers is a significant factor in determining the load-bearing capacity of FRC restorations. The resin matrix protects the fibers arrangement to provide ideal strengthening. The increased total fiber content in group III reduced the composite matrix volume and hence it showed less load-bearing capacity. Majority of the failures in group I were observed at the tooth-restoration interface. This was in accordance with the previous studies [35, 36]. In the restored teeth reinforced with fiber in the central part of the palatal surface, most of the fractures occurred in the natural tooth. This could explain the high strength of the fiber-reinforced composite, which exceeds the load-bearing capacity of the tooth, especially in the teeth with thin roots. The palatal anchorage from the high-strength fibers lessened the stress at the restoration-tooth junction. In the restorations reinforced with fibers at two positions, most failures were seen in the restored edges i.e. cohesive fracture. This might be the effect of fiber volume that may reduce the content and strength of the composite. It appears that higher fiber volume in the composite restoration may negatively affect its mechanical properties. A different pattern of fractures of restorations was reported by various researchers [37–41]. These differences may partly be explained by differences in the loading technique. FRC augments not only the impact strength and elasticity but also reduces the microleakage of composite resins [42–44]. To withstand greater impact forces during re-trauma conditions, the restorative material should possess higher fracture resistance ideally [24, 45, 46]. It is important to note that the addition of fibers in the composite restoration should not disturb the occlusion, otherwise possibilities of temporomandibular disorders are very high [26, 27, 47, 48].

In this in vitro study, intraorally produced forces in varied directions and intensities cannot be adequately replicated. The study does not consider the influence of thermocycling on fracture propagation characteristics. Here, fiber and composite resin only from a single company were assessed. Further assessment of the effect of thermo-mechanical stresses on the longevity of the restoration should be done. Various clinical trials are mandatory before using this technology routinely.

## Conclusion

Within the confines of our study, it can be concluded that the addition of FRC to the conventional incisal composite build-up increased the overall strength restoration. Such composite restoration reinforced with a single fiber on the palatal side showed the highest load-bearing capacity compared to two fibers reinforced and unreinforced composites. The common mode of failure in unreinforced composite was an adhesive failure, in two fibers reinforced composite was a cohesive failure, and in one fiber reinforced composite was in the remaining part of the tooth. The structural arrangement of FRC not only permits its closed adaption to the outlines of a tooth but also minimizes the chances of the fiber displacement during handling. Moreover, FRC is a biocompatible, esthetic, translucent material that disappears within the composite, thus improving the esthetic outcome which is primary requirement when selecting any restorative material for anterior teeth. Future studies should be planned to simulate the masticatory cycle considering cyclic and shear forces.

## Data Availability

The data is available with the corresponding author on request.

## Abbreviations

FRC: fiber-reinforced composite
IEC: Institutional Ethical Committee (),
LED: light-emitting diode
ANOVA: Analysis of variance
